# Microbiology and Management of Pediatric Liver Abscesses: Two Cases Caused by *Streptococcus anginosus* Group

**DOI:** 10.1155/2012/685953

**Published:** 2012-10-11

**Authors:** Michael Cellucci, Erin Simon, Stephen Eppes

**Affiliations:** ^1^Diagnostic Referral Division, Nemours/Alfred I. duPont Hospital for Children, 1600 Rockland Road, Wilmington, De 19803, USA; ^2^Nemours/Alfred I. duPont Hospital for Children, Thomas Jefferson University, USA; ^3^Division of Pediatric Infectious Disease, Nemours/AIfred I. duPont Hospital for Children, 1600 Rockland Road, Wilmington, De 19803, USA

## Abstract

Pyogenic liver abscesses in the pediatric population are rare occurrences in the developed world. We present two cases of previously healthy males presenting with fever and abdominal pain found to have liver abscesses due to organisms in the *Streptococcus anginosus* group. The microbiology of *S. anginosus* along with the management and recommended treatment in children with liver abscesses is discussed.

## 1. Case 1

**Figure 1:**
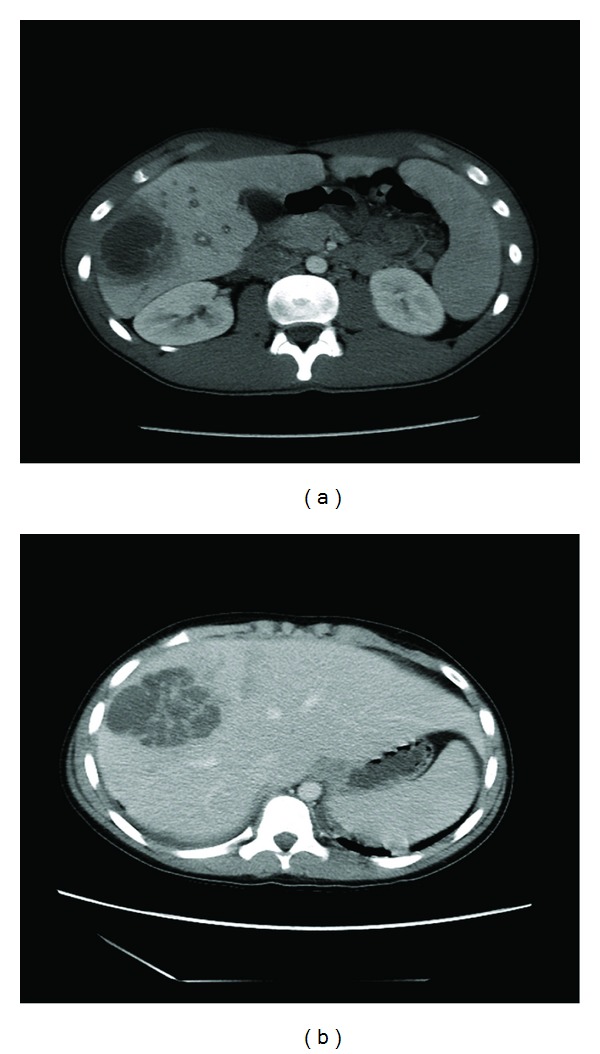
(a) Cystic lesion in the inferior right lobe of the liver that was rim enhancing and diffuse surrounding hypoattenuation. The cystic changes were measured to be 5.1 × 3.8.4.4 cm, along with heterogeneous appearance of the liver parenchyma suggestive of abscess. (b) Complex low-attenuation lesion in the right hepatic lobe with central irregular enhancement similar to liver and evidence of vascular or tumor thrombus in the middle hepatic vein. Additional low-attenuation foci were seen in the left and right lobes.

DK is a 16-year-old male presenting with severe abdominal pain. Three days prior while playing baseball, DK slid and began complaining of chest pain. Three days later he developed fever to 102°F and chills. His vital signs on presentation were blood pressure 102/71, pulse 108 bpm, respirations 16 bpm, and temperature 39.2°C. He was awake, tired, and flushed appearing. He had pain along his ribs on the lower right side of his abdomen along with decreased bowel sounds and tenderness of the right upper quadrant with voluntary guarding. There was no hepatosplenomegaly or masses. Labs included a disseminated intravascular coagulation (DIC) profile which showed fibrinogen 766 and a CBC showing WBC 13.6, HgB 12.7, Hct 35, platelets of 386 with 76% neutrophils, 15% lymphocytes, and 8% monocytes. Liver function testing was normal. He had a negative monospot, a normal amylase and lipase, an erythrocyte sedimentation rate (ESR) of 97 mm/hr, and a c-reactive protein (CRP) of 37.1 mg/dL. An ultrasound of the abdomen revealed a hypoechoic mass within the anterolateral right hepatic lobe further delineated by CT (Figure 1(a)) which revealed an abscess. He was placed on intravenous Piperacillin/Tazobactam followed by percutaneous drainage. Culture revealed 2+ *Streptococcus intermedius* (viridians strep). DK had serial CRPs which improved. A repeat ultrasound showed no lesion and the drain was removed. He was discharged home on intravenous Clindamycin; however, five days later he became febrile and returned to the emergency room. He had a WBC 17.7 with 85% neutrophils, a mild transaminitis, and a CRP of 5.8 mg/dL. An ultrasound of his abdomen revealed 4 × 2 cm fluid collection. He was taken to interventional radiology for repeat aspiration and replacement of drain. A follow-up ultrasound showed a persistent hypoechoic area adjacent to the drain consistent with hematoma so the drain was capped. Repeat ultrasound remained unchanged and the drain was removed. Inflammatory markers continued to decline and he was discharged home on intravenous ampicillin.

## 2. Case 2

MM is a 14-year-old male with cold symptoms four days prior to presentation followed by the onset of several episodes of emesis and diarrhea along with decreased appetite and fever to 105.4°F. His vital signs were blood pressure 99/47 mmHg, pulse 137, respirations 18, and temperature 39.5°C. He was well appearing with dry mucus membranes. His abdominal exam was benign without hepatosplenomegaly or masses. Labs were drawn, that showed a WBC 4.1, a hemoglobin 10.7 g/dL, platelets 109 K/uL, AST 95 U/L, ALT 93 U/L, albumin 2.7 g/dL, total and direct bilirubin 2.6 mg/dL and 0.6 mg/dL, respectively, PT 13.8 sec, and fibrinogen 744 mg/dL. During admission MM began complaining of RUQ abdominal pain and an ultrasound showed a 6 cm mass in the liver along with a thickened gallbladder wall. A CT of the abdomen was completed (Figure 1(b)) and a liver biopsy was performed with 3 mLs of purulent fluid drained. MM's blood culture and abscess culture revealed *Streptococcus consellatus* and he was started on intravenous Piperacillin/Tazobactam. A repeat ultrasound was performed five days later which showed interval increased size and cystic component of the liver abscess. He underwent repeat drainage and percutaneous drain placement. He had significant improvement and ultrasounds showed interval decrease in size and cystic components of the complex liver abscess. The drain was capped and a subsequent ultrasound revealed an interval decrease in size. The drain was then removed and he was discharged home on intravenous ceftriaxone. 

## 3. Discussion

Most descriptions of pyogenic liver abscesses (PLA) have been in the adult literature. These abscesses tend to be polymicrobial and typically are associated with cholangitis. PLA are a rare occurrence in infants and children, with an incidence of 0.007% to 0.04% of all hospital admissions per year. When left untreated, mortality rates of 80%–100% have been seen [1]. Most cases of liver abscesses result from direct extension from either the biliary or intestinal tract or from hematogenous spread. While both of our cases were previously healthy, the majority of cases of PLA have been described in patients in developing countries or those who are immunocompromised, especially those with chronic granulomatous disease (CGD) [2]. The clinical features of features of PLA are nonspecific: fever (89.6%), chills (69%), and abdominal pain (72.2%). However, only about 30% of patients present with all three symptoms [3]. Other common symptoms include nausea, vomiting, anorexia, weight loss, and malaise. Tender hepatomegaly has been described in adults but is rare in children [2]. Laboratory abnormalities include a leukocytosis (84% of patients), anemia (88.9% of patients), hypoalbuminemia (94% of patients), and an elevated alkaline phosphatase (73% of patients). An elevated CRP is commonly seen as well [2–4].

PLA are almost always the result of bacterial infections, although fungal infections may occur. *Entamoeba histolytica* is an important cause to consider in developing countries. Unlike adults, the most common organism identified in the pediatric population is *Staphylococcus aureus* but many organisms have been implicated which include Gram-negative bacteria such as *E. coli, Klebsiella,* and *Pseudomonas, anaerobes* and *streptococci*. The *streptococci* that were isolated from our patients were members of the *Streptococcus anginosus* group (also known as the *S. milleri* group) which is a subgroup of *Viridans Streptococci* that consists of three distinct streptococcal species: *S. anginosus, S. intermedius,* and *S. constellatus.* Members of the *S. anginosus* group are located in the intestinal tract and are the cause of many infections within the abdominal cavity [5, 6]. The *S. anginosus* group has a tendency to form abscess; however, the reasons are not completely understood. The group has been shown to possess intrinsic factors that are likely to be involved in their pathogenesis such as adhesins on their cell surfaces that facilitate adherence to cell walls and allow pathogens to attach to the sites of tissue damage [5, 6]. Members possess polysaccharide capsules that inhibit phagocytosis and enables them to replicate after arriving at and adhering to a site of tissue damage. In particular, *S. intermedius* produces a cytolytic exotoxin, intermedilysin, which has been noted to have potent hemolytic effects on human erythrocytes. The most likely virulence factor is due to the intermedilysin's production of superantigens which share the ability to activate specific lymphocyte subsets without regard to the antigenic specificity of the T cells and without prior cellular processing. After stimulation, superantigen-responsive T cells often die through apoptosis leading to acute toxic shock syndromes, necrotizing fasciitis, and multisystem illnesses due to the release of inflammatory cytokines [5]. These organisms tend to be sensitive to penicillin as was the case with both of our patients.

The early recognition of PLA is important. Abdominal ultrasound is the imaging modality of choice as it is diagnostic in over 90% of cases [3]. The right lobe of the liver is most commonly affected. When the abscesses are too small to be effectively drained (typically less than 2 cm) then one must employ a treatment strategy of antibiotics alone. In abscesses that are not multiloculated and less than 5 cm in size percutaneous needle aspiration and antibiotics have been described to be successful in most patients [4, 7]. Half of patients who undergo aspiration may need repeat aspiration when no indwelling drain is placed. This may need to be done up to three times [7, 8]. This approach of repeated aspiration without complete drainage in addition to antibiotics has been reported to be successful in 97% of patients [8]. In patients that fail this approach after 72 hours percutaneous drain placement is typically attempted under CT or sonographic guidance. Routine flushing of the drains is performed. Drains are left in place until the drainage is minimal. Risk factors for failed initial nonoperative management include multiloculated abscesses, biliary communication, increased serum urea and creatinine, or increased serum bilirubin [1]. Open laparotomy is typically reserved for those that display a poor response to percutaneous drainage and antibiotic therapy, when the pus is thick, or in patients with CGD [4]. Previously, laparotomy was also used in patients with multiple abscesses or those with biliary communication. Newer literature suggests that percutaneous drain placement is typically successful even in those with multiloculated abscesses, with multiple abscesses, or with biliary communication without obstruction and can be attempted in place of initial laparotomy [9]. 

Appropriate initial antibiotic regimens include ampicillin-sulbactam, Piperacillin-Tazobactam, or a third generation cephalosporin with metronidazole [1]. Antibiotic choices can be tailored once an organism is identified and susceptibilities are available. In immunocompromised patients, especially those with CGD, it is not unreasonable to add antifungal coverage [1, 2]. The recommended duration of treatment is 4–6 weeks in patient with multiple abscesses or shorter in those that have been well drained. Initial IV antibiotic therapy may be changed to appropriate oral antibiotic therapy after 2–4 weeks of treatment. One of the above regimens should be begun once a diagnosis of PLA is made, independent of other treatment plans. Monitoring response to treatment typically involves following serial inflammatory markers such as CRP. The clinical response may be delayed because of the time necessary to obtain effective concentrations in the liver. The median time to resolution of CRP is about 3 weeks [2]. Resolution on imaging typically lags behind clinical and laboratory improvement as some studies suggest sonographic evidence can persist for an average of 14 weeks and up to 2 years [10].
